# 3-[(2*E*)-2-(Butan-2-yl­idene)hydrazin­yl]-6-chloro­pyridazine

**DOI:** 10.1107/S160053681003504X

**Published:** 2010-09-04

**Authors:** Abdul Qayyum Ather, M. Nawaz Tahir, Misbahul Ain Khan, Muhammad Makshoof Athar

**Affiliations:** a Department of Chemistry, Islamia University, Bahawalpur, Pakistan; b Applied Chemistry Research Center, PCSIR Laboratories Complex, Lahore 54600, Pakistan; c University of Sargodha, Department of Physics, Sargodha, Pakistan; d Institute of Chemistry, University of the Punjab, Lahore, Pakistan

## Abstract

The asymmetric unit of the title compound, C_8_H_11_ClN_4_, contains two independent mol­ecules (*A* and *B*) with slightly different conformations: the dihedral angles between the 3-chloro-6-hydrazinylpyridazine units and butyl side chains are 4.5 (2) and 11.98 (16)°. In the crystal, the *A* and *B* mol­ecules are linked by a pair of N—H⋯N hydogen bonds, generating an *R*_2_^2^(8) loop.

## Related literature

For related structures, see: Ather *et al.* (2009 ▶, 2010 ▶). For graph-set notation, see: Bernstein *et al.* (1995 ▶).
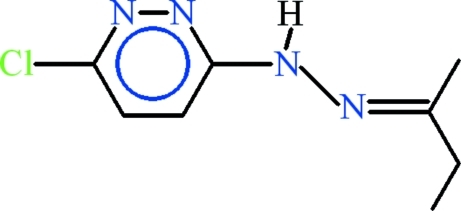


## Experimental

### 

#### Crystal data

C_8_H_11_ClN_4_
*M* *_r_* = 198.66
Triclinic, 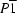

*a* = 8.0623 (4) Å
*b* = 11.6768 (5) Å
*c* = 12.1314 (5) Å
α = 113.858 (1)°
β = 91.370 (2)°
γ = 104.880 (2)°
*V* = 998.85 (8) Å^3^
*Z* = 4
Mo *K*α radiation
μ = 0.34 mm^−1^
*T* = 296 K
0.25 × 0.15 × 0.14 mm

#### Data collection

Bruker Kappa APEXII CCD diffractometer
Absorption correction: multi-scan (*SADABS*; Bruker, 2005 ▶) *T*_min_ = 0.982, *T*_max_ = 0.988
14983 measured reflections
3585 independent reflections
2652 reflections with *I* > 2σ(*I*)
*R*_int_ = 0.031

#### Refinement

*R*[*F* ^2^ > 2σ(*F* ^2^)] = 0.035
*wR*(*F* ^2^) = 0.099
*S* = 1.05
3585 reflections
239 parameters
H-atom parameters constrained
Δρ_max_ = 0.15 e Å^−3^
Δρ_min_ = −0.20 e Å^−3^

### 

Data collection: *APEX2* (Bruker, 2009 ▶); cell refinement: *SAINT* (Bruker, 2009 ▶); data reduction: *SAINT*; program(s) used to solve structure: *SHELXS97* (Sheldrick, 2008 ▶); program(s) used to refine structure: *SHELXL97* (Sheldrick, 2008 ▶); molecular graphics: *ORTEP-3 for Windows* (Farrugia, 1997 ▶) and *PLATON* (Spek, 2009 ▶); software used to prepare material for publication: *WinGX* (Farrugia, 1999 ▶) and *PLATON*.

## Supplementary Material

Crystal structure: contains datablocks global, I. DOI: 10.1107/S160053681003504X/hb5623sup1.cif

Structure factors: contains datablocks I. DOI: 10.1107/S160053681003504X/hb5623Isup2.hkl

Additional supplementary materials:  crystallographic information; 3D view; checkCIF report

## Figures and Tables

**Table 1:** Hydrogen-bond geometry (Å, °)

*D*—H⋯*A*	*D*—H	H⋯*A*	*D*⋯*A*	*D*—H⋯*A*
N3—H3*A*⋯N6^i^	0.86	2.30	3.0674 (15)	148
N7—H7⋯N2^i^	0.86	2.24	3.0689 (15)	161

Symmetry code: (i) 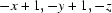.

**Fig. 1.**
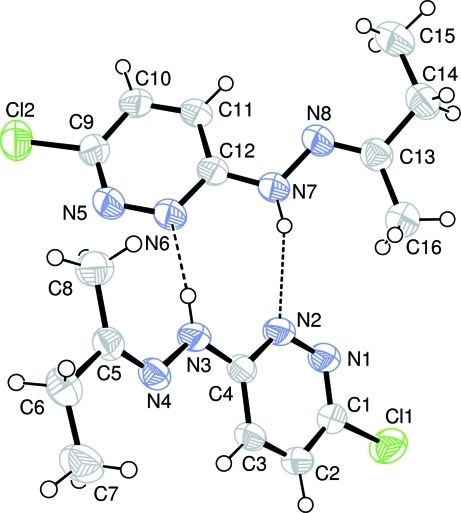
Two independent molecules of (I) with 50% probability displacement ellipsoids. Dashed lines denote intermolecular hydrogen bonds, forming a dimer: the N6 moleucle shown is generated by the symmetry operation (1–x, 1–y, –z) from the asymmetric atoms.

## supplementary crystallographic information

### Comment

In continuation of our studies of pyrazolylpyridazine derivatives (Ather *et al.*, 2009, 2010), the title compound (I, Fig. 1) is being reported here.

The title compound (I), consists of two independent molecules. In one molecule, the 3-chloro-6-hydrazinylpyridazine moiety A (C1—C4/N1—N4/CL1) and the butane group B (C5—C8) is planar with r. m. s. deviation of 0.0217 and 0.0130 Å. The dihedral angle between A/B is 4.53 (24)°. In second molecule, the 3-chloro-6-hydrazinylpyridazine moiety C (C9—C12/N5—N8/CL2) and the butane group D (C13—C16) is planar with r. m. s. deviation of 0.0453 and 0.0446 Å. The dihedral angle between C/D is 11.98 (16)°. The title compound consists of dimers due to N—H···N type of H-bonding (Table 1, Fig. 2) with *R*_2_^2^(8) ring motif (Bernstein *et al.*, 1995).

### Experimental

3-Chloro-6-hydrazinylpyridazine (0.5 g, 3.46 mmol), dissolved in ethyl-methylketone was refluxed for 30 min. The unreacted ethyl-methylketone was distilled off yielding the crude material. The product was re-crystallized in alcohol to affoard colorless needles of (I).

### Refinement

The H-atoms were positioned geometrically (N–H = 0.86, C–H = 0.93–0.97 Å) and were included in the refinement in the riding model approximation, with *U*_iso_(H) = x*U*_eq_(C, N), where x = 1.5 for methyl and x = 1.2 for all other H-atoms.

### Crystal data

**Table:**

C_8_H_11_ClN_4_	*Z* = 4
*M**_r_* = 198.66	*F*(000) = 416
Triclinic, *P*1	*D*_x_ = 1.321 Mg m^−^^3^
Hall symbol: -P 1	Mo *K*α radiation, λ = 0.71073 Å
*a* = 8.0623 (4) Å	Cell parameters from 2652 reflections
*b* = 11.6768 (5) Å	θ = 2.1–25.3°
*c* = 12.1314 (5) Å	µ = 0.34 mm^−^^1^
α = 113.858 (1)°	*T* = 296 K
β = 91.370 (2)°	Needle, colorless
γ = 104.880 (2)°	0.25 × 0.15 × 0.14 mm
*V* = 998.85 (8) Å^3^	

### Data collection

**Table:**

Bruker Kappa APEXII CCD diffractometer	3585 independent reflections
Radiation source: fine-focus sealed tube	2652 reflections with *I* > 2σ(*I*)
graphite	*R*_int_ = 0.031
Detector resolution: 8.10 pixels mm^-1^	θ_max_ = 25.3°, θ_min_ = 2.1°
ω scans	*h* = −9→9
Absorption correction: multi-scan (*SADABS*; Bruker, 2005)	*k* = −13→13
*T*_min_ = 0.982, *T*_max_ = 0.988	*l* = −14→11
14983 measured reflections	

### Refinement

**Table:**

Refinement on *F*^2^	Primary atom site location: structure-invariant direct methods
Least-squares matrix: full	Secondary atom site location: difference Fourier map
*R*[*F*^2^ > 2σ(*F*^2^)] = 0.035	Hydrogen site location: inferred from neighbouring sites
*wR*(*F*^2^) = 0.099	H-atom parameters constrained
*S* = 1.05	*w* = 1/[σ^2^(*F*_o_^2^) + (0.0426*P*)^2^ + 0.1867*P*] where *P* = (*F*_o_^2^ + 2*F*_c_^2^)/3
3585 reflections	(Δ/σ)_max_ = 0.001
239 parameters	Δρ_max_ = 0.15 e Å^−^^3^
0 restraints	Δρ_min_ = −0.20 e Å^−^^3^

### Special details

**Table:**

Geometry. Bond distances, angles *etc*. have been calculated using the rounded fractional coordinates. All su's are estimated from the variances of the (full) variance-covariance matrix. The cell e.s.d.'s are taken into account in the estimation of distances, angles and torsion angles
Refinement. Refinement of *F*^2^ against ALL reflections. The weighted *R*-factor *wR* and goodness of fit *S* are based on *F*^2^, conventional *R*-factors *R* are based on *F*, with *F* set to zero for negative *F*^2^. The threshold expression of *F*^2^ > σ(*F*^2^) is used only for calculating *R*-factors(gt) *etc*. and is not relevant to the choice of reflections for refinement. *R*-factors based on *F*^2^ are statistically about twice as large as those based on *F*, and *R*- factors based on ALL data will be even larger.

### Fractional atomic coordinates and isotropic or equivalent isotropic displacement parameters (Å^2^)

**Table:**

	*x*	*y*	*z*	*U*_iso_*/*U*_eq_	
Cl1	0.56878 (7)	1.01144 (5)	0.14461 (4)	0.0711 (2)	
N1	0.65078 (7)	0.81818 (4)	−0.01826 (4)	0.0494 (5)	
N2	0.69138 (9)	0.70411 (6)	−0.06328 (4)	0.0477 (5)	
N3	0.73568 (11)	0.52631 (8)	−0.04789 (4)	0.0485 (5)	
N4	0.75811 (18)	0.46292 (14)	0.02371 (12)	0.0446 (5)	
C1	0.6218 (2)	0.86549 (17)	0.09480 (15)	0.0457 (6)	
C2	0.6309 (2)	0.80724 (18)	0.17388 (15)	0.0482 (6)	
C3	0.6692 (2)	0.69257 (17)	0.12939 (14)	0.0456 (6)	
C4	0.6972 (2)	0.64155 (16)	0.00695 (14)	0.0403 (6)	
C5	0.8018 (2)	0.35764 (18)	−0.02463 (16)	0.0446 (6)	
C6	0.8294 (3)	0.29374 (19)	0.05683 (17)	0.0546 (7)	
C7	0.8037 (3)	0.3631 (2)	0.18693 (18)	0.0706 (9)	
C8	0.8324 (3)	0.29472 (19)	−0.15401 (16)	0.0571 (7)	
Cl2	0.61890 (8)	0.92216 (5)	0.55956 (5)	0.0741 (2)	
N5	0.4684 (2)	0.70148 (16)	0.37821 (14)	0.0577 (6)	
N6	0.3743 (2)	0.57428 (16)	0.32230 (13)	0.0577 (6)	
N7	0.2290 (2)	0.38112 (15)	0.32596 (13)	0.0557 (6)	
N8	0.1391 (2)	0.31693 (16)	0.38987 (13)	0.0527 (6)	
C9	0.4960 (2)	0.75931 (18)	0.49629 (16)	0.0499 (6)	
C10	0.4326 (3)	0.69965 (19)	0.57130 (16)	0.0572 (7)	
C11	0.3386 (3)	0.57264 (19)	0.51703 (16)	0.0560 (7)	
C12	0.3135 (2)	0.51037 (18)	0.38917 (15)	0.0461 (6)	
C13	0.0646 (3)	0.19453 (19)	0.33475 (16)	0.0514 (7)	
C14	−0.0380 (3)	0.1305 (2)	0.40720 (18)	0.0656 (8)	
C15	−0.0180 (3)	0.2152 (2)	0.5419 (2)	0.0842 (10)	
C16	0.06718 (9)	0.10973 (7)	0.20380 (5)	0.0690 (8)	
H2	0.61145	0.84592	0.25415	0.0578*	
H3	0.67676	0.64881	0.17770	0.0547*	
H3A	0.74569	0.49409	−0.12405	0.0582*	
H6A	0.75051	0.20566	0.02287	0.0655*	
H6B	0.94655	0.28638	0.05598	0.0655*	
H7A	0.88583	0.44865	0.22347	0.1059*	
H7B	0.68809	0.37098	0.18942	0.1059*	
H7C	0.82074	0.31382	0.23104	0.1059*	
H8A	0.72987	0.27583	−0.20780	0.0858*	
H8B	0.92751	0.35314	−0.16869	0.0858*	
H8C	0.85936	0.21485	−0.16845	0.0858*	
H7	0.23145	0.34080	0.24923	0.0668*	
H10	0.45400	0.74553	0.65567	0.0687*	
H11	0.29214	0.52778	0.56265	0.0673*	
H14A	−0.00369	0.05316	0.39609	0.0787*	
H14B	−0.15974	0.10162	0.37404	0.0787*	
H15A	0.10043	0.23887	0.57731	0.1264*	
H15B	−0.09207	0.16776	0.57958	0.1264*	
H15C	−0.04957	0.29300	0.55442	0.1264*	
H16A	−0.00022	0.13112	0.15265	0.1035*	
H16B	0.01880	0.01944	0.18790	0.1035*	
H16C	0.18459	0.12428	0.18693	0.1035*	

### Atomic displacement parameters (Å^2^)

**Table:**

	*U*^11^	*U*^22^	*U*^33^	*U*^12^	*U*^13^	*U*^23^
Cl1	0.1018 (5)	0.0565 (3)	0.0605 (3)	0.0378 (3)	0.0197 (3)	0.0210 (3)
N1	0.0617 (10)	0.0490 (9)	0.0437 (8)	0.0192 (8)	0.0131 (7)	0.0235 (7)
N2	0.0627 (10)	0.0477 (9)	0.0393 (8)	0.0201 (8)	0.0144 (7)	0.0222 (7)
N3	0.0670 (10)	0.0479 (9)	0.0362 (7)	0.0190 (8)	0.0112 (7)	0.0219 (7)
N4	0.0485 (9)	0.0478 (9)	0.0427 (8)	0.0109 (7)	0.0063 (7)	0.0261 (7)
C1	0.0480 (11)	0.0428 (10)	0.0437 (10)	0.0106 (8)	0.0075 (8)	0.0173 (8)
C2	0.0534 (11)	0.0505 (11)	0.0360 (9)	0.0100 (9)	0.0095 (8)	0.0167 (8)
C3	0.0521 (11)	0.0494 (11)	0.0367 (9)	0.0091 (9)	0.0078 (8)	0.0230 (8)
C4	0.0404 (10)	0.0420 (10)	0.0373 (9)	0.0066 (8)	0.0055 (7)	0.0188 (8)
C5	0.0414 (10)	0.0454 (11)	0.0467 (10)	0.0070 (8)	0.0060 (8)	0.0228 (9)
C6	0.0529 (12)	0.0582 (12)	0.0621 (12)	0.0144 (10)	0.0074 (9)	0.0358 (10)
C7	0.0867 (16)	0.0831 (16)	0.0590 (12)	0.0271 (13)	0.0127 (11)	0.0454 (12)
C8	0.0663 (13)	0.0581 (12)	0.0506 (11)	0.0227 (10)	0.0131 (9)	0.0236 (10)
Cl2	0.0911 (4)	0.0514 (3)	0.0707 (4)	0.0066 (3)	0.0057 (3)	0.0259 (3)
N5	0.0740 (12)	0.0536 (10)	0.0461 (9)	0.0099 (9)	0.0093 (8)	0.0269 (8)
N6	0.0790 (12)	0.0537 (10)	0.0401 (8)	0.0104 (9)	0.0088 (8)	0.0249 (8)
N7	0.0760 (11)	0.0497 (10)	0.0379 (8)	0.0079 (8)	0.0112 (8)	0.0213 (7)
N8	0.0610 (10)	0.0546 (10)	0.0466 (9)	0.0123 (8)	0.0115 (7)	0.0282 (8)
C9	0.0580 (12)	0.0459 (11)	0.0482 (10)	0.0162 (9)	0.0093 (9)	0.0215 (9)
C10	0.0795 (14)	0.0535 (13)	0.0373 (10)	0.0183 (11)	0.0123 (9)	0.0185 (9)
C11	0.0786 (14)	0.0534 (12)	0.0409 (10)	0.0169 (11)	0.0182 (9)	0.0256 (9)
C12	0.0540 (11)	0.0488 (11)	0.0394 (9)	0.0147 (9)	0.0086 (8)	0.0225 (9)
C13	0.0534 (12)	0.0540 (12)	0.0493 (10)	0.0128 (10)	0.0069 (9)	0.0261 (10)
C14	0.0692 (14)	0.0642 (14)	0.0642 (13)	0.0077 (11)	0.0132 (10)	0.0349 (11)
C15	0.1004 (19)	0.0848 (18)	0.0671 (15)	0.0104 (14)	0.0291 (13)	0.0410 (13)
C16	0.0818 (16)	0.0572 (13)	0.0572 (12)	0.0078 (11)	0.0125 (11)	0.0212 (10)

### Geometric parameters (Å, °)

**Table:**

Cl1—C1	1.734 (2)	C6—H6B	0.9700
Cl2—C9	1.733 (2)	C6—H6A	0.9700
N1—N2	1.3504 (9)	C7—H7A	0.9600
N1—C1	1.3075 (17)	C7—H7C	0.9600
N2—C4	1.3345 (19)	C7—H7B	0.9600
N3—N4	1.3843 (18)	C8—H8A	0.9600
N3—C4	1.360 (2)	C8—H8C	0.9600
N4—C5	1.276 (3)	C8—H8B	0.9600
N3—H3A	0.8600	C9—C10	1.388 (3)
N5—C9	1.297 (2)	C10—C11	1.348 (3)
N5—N6	1.350 (3)	C11—C12	1.405 (2)
N6—C12	1.333 (3)	C13—C16	1.4981 (19)
N7—N8	1.381 (2)	C13—C14	1.505 (3)
N7—C12	1.357 (3)	C14—C15	1.508 (3)
N8—C13	1.272 (3)	C10—H10	0.9300
N7—H7	0.8600	C11—H11	0.9300
C1—C2	1.392 (3)	C14—H14A	0.9700
C2—C3	1.347 (3)	C14—H14B	0.9700
C3—C4	1.408 (2)	C15—H15A	0.9600
C5—C8	1.498 (3)	C15—H15B	0.9600
C5—C6	1.501 (3)	C15—H15C	0.9600
C6—C7	1.503 (3)	C16—H16A	0.9600
C2—H2	0.9300	C16—H16B	0.9600
C3—H3	0.9300	C16—H16C	0.9600
			
N2—N1—C1	118.43 (10)	H8A—C8—H8C	109.00
N1—N2—C4	119.67 (8)	C5—C8—H8A	109.00
N4—N3—C4	117.38 (10)	H8B—C8—H8C	109.00
N3—N4—C5	118.39 (13)	H8A—C8—H8B	109.00
C4—N3—H3A	121.00	C5—C8—H8C	109.00
N4—N3—H3A	121.00	C5—C8—H8B	109.00
N6—N5—C9	119.09 (18)	Cl2—C9—C10	119.98 (14)
N5—N6—C12	119.53 (15)	Cl2—C9—N5	115.69 (16)
N8—N7—C12	117.28 (14)	N5—C9—C10	124.3 (2)
N7—N8—C13	119.01 (15)	C9—C10—C11	117.37 (17)
N8—N7—H7	121.00	C10—C11—C12	117.67 (19)
C12—N7—H7	121.00	N7—C12—C11	122.28 (19)
N1—C1—C2	124.75 (16)	N6—C12—N7	115.75 (15)
Cl1—C1—C2	119.92 (13)	N6—C12—C11	121.96 (19)
Cl1—C1—N1	115.33 (13)	N8—C13—C14	116.84 (17)
C1—C2—C3	117.28 (16)	N8—C13—C16	125.68 (18)
C2—C3—C4	117.42 (17)	C14—C13—C16	117.47 (17)
N2—C4—N3	114.99 (12)	C13—C14—C15	115.48 (19)
N3—C4—C3	122.59 (16)	C9—C10—H10	121.00
N2—C4—C3	122.41 (16)	C11—C10—H10	121.00
C6—C5—C8	117.44 (18)	C10—C11—H11	121.00
N4—C5—C8	125.76 (19)	C12—C11—H11	121.00
N4—C5—C6	116.78 (16)	C13—C14—H14A	108.00
C5—C6—C7	115.54 (19)	C13—C14—H14B	108.00
C3—C2—H2	121.00	C15—C14—H14A	108.00
C1—C2—H2	121.00	C15—C14—H14B	108.00
C4—C3—H3	121.00	H14A—C14—H14B	107.00
C2—C3—H3	121.00	C14—C15—H15A	109.00
C5—C6—H6A	108.00	C14—C15—H15B	109.00
C7—C6—H6B	108.00	C14—C15—H15C	109.00
C5—C6—H6B	108.00	H15A—C15—H15B	110.00
H6A—C6—H6B	107.00	H15A—C15—H15C	109.00
C7—C6—H6A	108.00	H15B—C15—H15C	110.00
H7A—C7—H7B	110.00	C13—C16—H16A	109.00
H7A—C7—H7C	109.00	C13—C16—H16B	109.00
C6—C7—H7C	109.00	C13—C16—H16C	109.00
H7B—C7—H7C	109.00	H16A—C16—H16B	109.00
C6—C7—H7B	109.00	H16A—C16—H16C	109.00
C6—C7—H7A	109.00	H16B—C16—H16C	109.00
			
C1—N1—N2—C4	1.45 (15)	N8—N7—C12—C11	12.0 (3)
N2—N1—C1—Cl1	−179.47 (8)	N7—N8—C13—C16	1.2 (3)
N2—N1—C1—C2	0.5 (2)	N7—N8—C13—C14	−177.43 (18)
N1—N2—C4—N3	178.67 (10)	N1—C1—C2—C3	−1.3 (3)
N1—N2—C4—C3	−2.6 (2)	Cl1—C1—C2—C3	178.68 (14)
C4—N3—N4—C5	−176.95 (15)	C1—C2—C3—C4	0.2 (3)
N4—N3—C4—N2	174.58 (12)	C2—C3—C4—N2	1.8 (3)
N4—N3—C4—C3	−4.1 (2)	C2—C3—C4—N3	−179.62 (16)
N3—N4—C5—C6	178.44 (15)	N4—C5—C6—C7	−0.6 (3)
N3—N4—C5—C8	0.2 (3)	C8—C5—C6—C7	177.78 (19)
C9—N5—N6—C12	−0.8 (3)	Cl2—C9—C10—C11	−179.66 (18)
N6—N5—C9—Cl2	179.70 (14)	N5—C9—C10—C11	1.1 (3)
N6—N5—C9—C10	−1.0 (3)	C9—C10—C11—C12	0.6 (3)
N5—N6—C12—N7	−176.31 (16)	C10—C11—C12—N6	−2.3 (3)
N5—N6—C12—C11	2.5 (3)	C10—C11—C12—N7	176.4 (2)
C12—N7—N8—C13	−176.99 (19)	N8—C13—C14—C15	−8.9 (3)
N8—N7—C12—N6	−169.24 (16)	C16—C13—C14—C15	172.35 (18)

### Hydrogen-bond geometry (Å, °)

**Table:**

*D*—H···*A*	*D*—H	H···*A*	*D*···*A*	*D*—H···*A*
N3—H3A···N6^i^	0.86	2.30	3.0674 (15)	148
N7—H7···N2^i^	0.86	2.24	3.0689 (15)	161

Symmetry codes: (i) −*x*+1, −*y*+1, −*z*.
